# Clinical Lectures on Surgery

**Published:** 1856-01

**Authors:** 


ART. VII.—*Clinical Lectures on Surgery.* By M. NÈLATON. From Notes taken by WALTER ATLEE, M. D. Philadelphia: J. B. Lippincott & Co. 1855.

M. Nèlaton is one of the Professors in the Clinical Hospital of the Faculty of Medicine, Paris. This volume is made up from notes taken during a three year's attendance on his lectures by the young author. It will be recollected that we have previously had a little volume from the same source, made up in the same manner from the lectures of Bernard and Robin.

The present is a more imposing effort, in the form of a goodly octavo of some 750 pages. It does not touch upon all the subjects of practical surgery; but, nevertheless, includes a large number of them. It has very much practical merit. The cases are well selected, and (as should be the case in all published clinical lectures) used as pegs upon which to hang general remarks on pathology and treatment, rather than as simple histories of cases. The style of description is clear and accurate, without being tediously minute; while the pathological portions of the work are very intelligent. All in all it is a very creditable book, and will form a most useful addition to the surgeon's library.

For sale by PHINNEY & Co.

